# Topical Application of 0.5% Timolol Maleate Hydrogel for the Treatment of Superficial Infantile Hemangioma

**DOI:** 10.3389/fonc.2017.00137

**Published:** 2017-06-27

**Authors:** Hai Wei Wu, Chao Liu, Xuan Wang, Ling Zhang, Weien Yuan, Jia Wei Zheng, Li Xin Su, Xin Dong Fan

**Affiliations:** ^1^Department of Oral and Maxillofacial Surgery, Shanghai Ninth People’s Hospital, College of Stomatology, Shanghai Jiao Tong University School of Medicine, Shanghai, China; ^2^Department of Oral and Maxillofacial Surgery, Shandong Provincial Hospital Affiliated to Shandong University, Jinan, Shandong, China; ^3^Department of General Dentistry, Shanghai Ninth People’s Hospital, College of Stomatology, Shanghai Jiao Tong University School of Medicine, Shanghai, China; ^4^School of Pharmacy, Shanghai Jiao Tong University, Shanghai, China; ^5^Department of Interventional Therapy, Shanghai Ninth People’s Hospital, College of Stomatology, Shanghai Jiao Tong University School of Medicine, Shanghai, China

**Keywords:** infantile hemangioma, timolol maleate, hydrogel, topical therapy, drug safety

## Abstract

The therapeutic options for infantile hemangiomas (IHs) have been greatly altered since the introduction of oral propranolol for successful treatments of IHs. Recently, there is an increase in the application of topical timolol maleate for treating superficial IHs. In the present study, we developed a new formulation of timolol maleate 0.5% hydrogel and treated 321 patients with superficial IHs to evaluate its efficacy and safety in the treatment of superficial IHs. This new timolol hydrogel was applied three times daily with a mean duration of 7.1 months. Response to treatment was assessed according to cosmetic improvement by using visual analog scale (VAS). The average VAS improvement after treatment was 76.4, with 126 patients (39.3%) achieving excellent responses, 159 patients (49.5%) achieving good responses, 33 patients (10.3%) achieving fair responses, and three patients (0.9%) achieving poor responses. Age at treatment initiation (*P* = 0.0349) and lesion thickness (*P* = 0.0147) were significantly associated with therapeutic efficacy. No severe side effects were observed in all patients. In conclusion, this new topical timolol maleate 0.5% hydrogel appears to be a proper candidate for treating superficial IHs, and our study provides supportive evidence and experience of topical timolol maleate in treating superficial IHs.

## Introduction

Infantile hemangiomas (IHs) are the most frequently occurring pediatric lesions, which are benign vascular tumors with a characteristic growth pattern of rapid proliferation and slow regression over several years (1). Data from present publications indicates that decreasing gestational age, low birth weight, and a female predominance are closely associated with higher IH incidence (2). The decision on the initiation of IH treatment has been controversial in the past years due to the unique growth pattern of IHs. However, recent studies showed that most untreated IHs did not improve after 3.5 years of age (3), and more than 50% of untreated IHs exhibited significant residual lesions (4, 5). Thus, early and active intervention instead of “wait and see” policy might be a better strategy for the treatment of IHs.

The therapeutic options for IHs have been greatly altered since the introduction of oral propranolol for successful treatment of IHs. Numerous clinical studies have proved the efficacy and safety of propranolol treatment, which is now recommended as the first-line therapy in treating IHs (6). However, there is still controversy over the potential side effects of propranolol because propranolol could easily penetrate the blood–brain barrier and might affect the development of central nervous system (7). To avoid potential systemic side effects, topical beta blockers have been applied in the treatment of IHs, especially for superficial lesions. Significant progress for transdermal drug delivery has been made, including microneedle transdermal patch (8–11) and nano- transdermal drug delivery (12). In our previous study, topical application of nano-propranolol 0.5% hydrogel achieved good efficacy in treating uncomplicated superficial IHs (12). Recently, there is an increase in the application of topical timolol maleate for treating superficial IHs (13). However, clinical studies based on large samples are very limited, and no consensus exists about the use of topical timolol for treating IHs (13). In the present study, we treated superficial IHs with a new topical timolol maleate 0.5% hydrogel and aimed to evaluate the efficacy and safety in the treatment of superficial IHs in a large case series.

## Materials and Methods

### Study Design

The study was conducted at Department of Oral and Maxillofacial Surgery, Shanghai Ninth People’s Hospital, College of Stomatology, Shanghai Jiao Tong University School of Medicine. The study population was composed of 321 consecutive patients with superficial IHs who received treatment between June 2014 and June 2016. The study was carried out in accordance with Declaration of Helsinki. Informed consents were obtained from all parents of the patients and the study was approved by the Institute Review Board of Shanghai Ninth People’s Hospital. The exclusion criteria included hypersensitivity to timolol maleate, heart defects, or arrhythmia, a history with previous treatment, ulcerated, mucosal, or subcutaneous IHs, lesion thickness >10 mm and treatment duration less than 2 months.

### Preparation of Novel Timolol Maleate 0.5% Hydrogel

Timolol maleate 0.5% hydrogel was prepared as follows: 16 g polyethylene glycol (PEG400, molecular weight: 400 Da), 5 g sodium benzoate, and 40 g glycerol dispersed in 200 ml purified water, and then 5 g timolol maleate was added into the well dispersed PVA hydrogel [32 g polyvinyl alcohol (PVA, molecular weight: 40,000 Da) and 32 g PVA (molecular weight: 100,000 Da)] dissolved in 670 ml purified water. The preparation of timolol maleate 0.5% hydrogel was stored in refrigerator at 4°C.

### Treatment Regimen

Baseline screening was completed based on the criteria reported by Chan et al. (14), and all patients underwent a thorough physical examination prior to treatment, including cardiovascular examination, ultrasound investigation, and clinical photography. 321 patients met the inclusion criteria and were enrolled in the study. The hydrogel was applied three times daily, gently rubbed as a thin layer onto the whole surface of IH. During the first application of the hydrogel, cardiovascular examination (including heart rate and blood pressure) was performed before and after the treatment. Then cardiovascular examination was performed every 1 month as a routine. Consistent with our previous study (15), drug withdrawal criterion was based on clinical responses. Drug administration was continued until objective goals were obtained or no further improvement was achieved. The application of topical timolol was tapered by decreasing to twice a day for 2 weeks followed by once a day for 2 weeks, and then discontinued.

### Outcome Measurement

The main goal of the treatment is to reduce cosmetic impairment caused by superficial lesions. Therefore, response to treatment was mainly assessed according to cosmetic improvement by using visual analog scale (VAS) (13, 16). The VAS score ranges from −100 (representing a doubling in the size and extent of the IH) to 100 (representing complete resolution), which was evaluated by another two independent physicians after reviewing all clinical photographs of IHs at baseline and after the full course of treatment. Therapeutic responses were graded as follows: excellent response (VAS score ranging from 90 to 100), good response (VAS score ranging from 50 to 90), fair response (VAS score ranging from 0 to 50), and poor response (VAS score ranging from −100 to 0). The follow-up period after the treatment ranged from 6 months to 1 year.

### Evaluation of Skin Permeation of Topical Timolol Maleate 0.5% Hydrogel and Gel

To briefly evaluate the skin permeation of different formulations of topical timolol, UV detection was applied to compare the residual drug concentration after topical application of commercially available timolol maleate 0.5% gel (Timoptic-XE, Aton Pharma, Inc.) or this new timolol maleate 0.5% hydrogel. Six patients with superficial IHs (>2 cm^2^) were treated with both timolol maleate 0.5% gel and timolol maleate 0.5% hydrogel simultaneously. 2 ml gel or hydrogel was rubbed as a thin layer onto 1 cm^2^ surface of IH in the same patient. All samples were collected at different time points (0, 4, 8, and 12 h after the treatment) and dissolved in 20 ml solution (ethanol: distilled water = 4:1) for detection of timolol maleate. The detector wavelength was adjusted to 296 nm according to the UV spectra of timolol (17) and optical density (OD) values of different groups were recorded. Permeation rate was calculated as follows: (OD values at 0 h − OD values at 4 h/8 h/12 h) × 100%/OD values at 0 h.

### Statistical Methods

Analysis was performed using the SPSS software package (version 16.0; SPSS, Chicago, IL, USA). Descriptive data were expressed as numbers, percentages, or means ± SDs. Mann–Whitney *U*-test was used to compare the clinical responses at different groups. *P*-values <0.05 were considered as significant.

## Results

### Clinical and Histological Features

The clinical characteristics of patients are listed in Table 1. Three hundred twenty-one patients were included in the study. The mean age at treatment initiation was 5.4 months, ranging from 2 to 21 months. There was a female predominance with a ratio of 2.7 to 1. The primary locations included head and neck region (58.9%), torso, and extremities (Table 1). Among all superficial IHs, there were 45.8% of lesions less than 1 mm in thickness and 54.2% more than 1 mm in thickness. Duration of topical timolol treatment ranged from 2 to 20 months, with a mean duration of 7.1 months.

**Table 1:** Clinical characteristics of patients.

Clinical characteristic	*n* (%)
**Age**	
Mean age (months)	5.4
Range (months)	2–21
**Gender**	
Male	87 (27.1)
Female	234 (72.9)
**Lesion location**	
Head and neck	189 (58.9)
Body	132 (41.1)
**Lesion thickness**	
≤1 mm in thickness	147 (45.8)
>1 mm in thickness	174 (54.2)
**Duration of treatment**	
Mean duration (months)	7.1
Range (months)	2–20

### Therapeutic Outcomes

**Figure 1:**
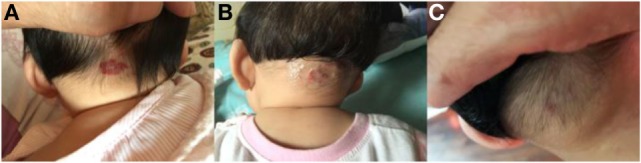
Response of superficial infantile hemangiomas in the nape to topical timolol maleate 0.5% hydrogel. **(A)** Before starting topical timolol therapy, at the age of 3 months; **(B)** 1 month after starting topical timolol therapy; **(C)** at the age of 6 months after end of topical timolol therapy; obvious discoloration and regression in size were noted.

**Figure 2:**
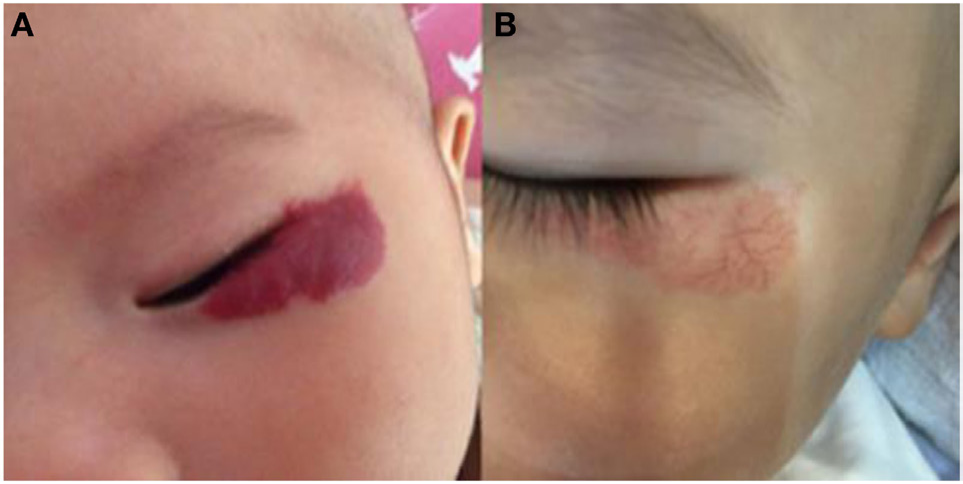
Response of superficial infantile hemangiomas in the periorbital area to topical timolol maleate 0.5% hydrogel. **(A)** Before starting topical timolol therapy, at the age of 2 months; **(B)** at the age of 14 months after end of topical timolol therapy; obvious discoloration was noted.

**Figure 3:**
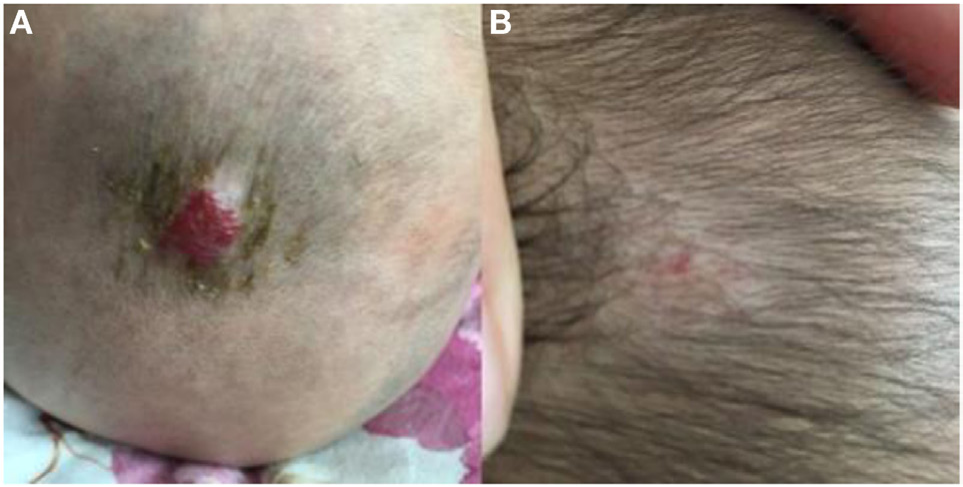
Response of superficial infantile hemangiomas in the scalp to topical timolol maleate 0.5% hydrogel. **(A)** Before starting topical timolol therapy, at the age of 5 months; **(B)** at the age of 13 months after end of topical timolol therapy; obvious discoloration and regression in size were noted.

The average VAS improvement after treatment was 76.4, with 126 patients (39.3%) achieving excellent responses, 159 patients (49.5%) achieving good responses, 33 patients (10.3%) achieving fair responses, and 3 patients (0.9%) achieving poor responses. Typical images with excellent responses are shown in Figures 1–3. Clinical responses demonstrated as blanching and softening of the lesions after treatment initiation. No patients experienced recurrence or rebound growth of the lesion at the last follow-up. Analysis of predictors of clinical responses is listed in Table 2, and clinical therapeutic differences in age at treatment initiation and duration of treatment is listed in Table 3. Age at treatment initiation (*P* = 0.0349) and lesion thickness (*P* = 0.0147) were associated with therapeutic efficacy. No significant VAS difference was observed in variables of gender, duration of treatment and lesion location.

**Table 2:** Analysis of predictors of clinical response.

Variables	Mean VAS ± SD	Average duration of treatment (months)	*P-*value
**Gender**			0.3154
Male	72.5 ± 25.6	7.4	
Female	72.8 ± 22.5	6.9	
**Age at treatment initiation**			0.0349*
≤6 months	81.3 ± 16.7	7.6	
>6 months	63.8 ± 32.2	5.9	
**Duration of treatment**			0.3691
≤6 months	73.3 ± 26.4	4.3	
>6 months	80.4 ± 18.3	10.9	
**Lesion location**			0.2487
Head and neck	72.3 ± 27.1	7.2	
Body	82.2 ± 15.5	6.8	
**Lesion thickness**			0.0147*
≤1 mm in thickness	81.7 ± 18.5	5.7	
>1 mm in thickness	70.1 ± 27.0	8.6	

*P < 0.05 was considered as significant.

*VAS, visual analog scale*.

**Table 3:** Clinical therapeutic differences in age at treatment initiation and duration of treatment.

Variables	Treatment responses			

	Excellent	Good	Fair	Poor
**Age**				
≤6 months	96	120	12	0
>6 months	30	39	21	3
**Duration**				
≤6 months	72	81	19	2
>6 months	54	78	14	1

### Complications

No systemic adverse effects were noted during treatment. Local side effects were observed in six patients, with four patients experiencing pruritus and two patients experiencing excoriations.

### Permeation Rate of Different Formulations of Topical Timolol Maleate

As shown in Table 4, permeation rate of novel topical timolol maleate 0.5% hydrogel was much higher than commercially available timolol maleate 0.5% gel 4, 8, and 12 h after administration.

**Table 4:** Permeation rate (%) of topical timolol maleate 0.5% hydrogel or gel.

Samples	Mean percentage ± SD at 4 h (%)	Mean percentage ± SD at 8 h (%)	Mean percentage ± SD at 12 h (%)
Topical timolol maleate 0.5% hydrogel	25.5 ± 9.4	35.1 ± 7.2	47.6 ± 9.4
Topical timolol maleate 0.5% gel	17.1 ± 8.0	21.8 ± 10.5	25.5 ± 11.0

## Discussion

Infantile hemangiomas are classified into superficial, deep, and compound types according to lesion distribution and anatomic depth of involvement (18). Oral propranolol has been the first-line treatment for high-risk and deep IH, and topical beta blocker therapy is recently attempted for the treatment of superficial IHs. Although topical beta blockers have been widely used for superficial IHs with acceptable effects, there is no consensus on the choice of beta blockers, optimal drug concentration, treatment frequency, and duration of treatment. In the present study, we applied a new timolol maleate 0.5% hydrogel three times daily with a mean duration of 7.1 months for the treatment of superficial IHs. Clinical efficacy was observed in all but three patients, and no one experienced severe systemic side effects. This study provided supportive evidence of the efficacy and safety of timolol in treating superficial IHs.

Currently, topical drug therapy for superficial IHs has been prepared in several ways, including corticosteroids cream, imiquimod 5% cream, propranonlol 0.5% hydrogel, propranolol 1% ointment, propranolol 2% ointment, propranolol 3% hydrogel, timolol 0.1% gel, timolol 0.25% gel forming solution, timolol 0.5% eye drop, timolol 0.5% gel forming solution, and timolol 0.5% gel and so on (19). In the present study, we developed a new formulation of timolol maleate 0.5% hydrogel. Timolol maleate was incorporated into PEG/PVA hydrogel, which has become candidate nano materials for controlled drug delivery (20). UV detection of timolol maleate demonstrated that this novel hydrogel could permeate skin more easily than commercially available timolol maleate 0.5% gel with the help of PEG/PVA hydrogel system, suggesting higher bioavailability and better drug percutaneous absorption of the new hydrogel. The hydrogel can keep skin moist and coating, which could reduce cutaneous stimulus of the topical drug and incidence of side effects. Compared with timolol eye drop and gel forming solution, the hydrogel was more convenient to apply in infants because solutions were easily flowing while the hydrogel would immediately become a thin layer of gel after application.

Few clinical studies were implemented to compare the efficacy and safety of different drugs. In a retrospective study by Qiu et al., timolol 0.5% eye drop and imiquimod 5% cream achieved equivalent clinical efficacy, but fewer side effects were observed in patients treating with topical timolol (16). Danarti et al. compared the effectiveness of topical corticosteroids and timolol maleate 0.5% solution or gel (21). Effectiveness of topical timolol maleate 0.5% solution or gel was significantly better than topical corticosteroids, but there was no difference in IHs treating with timolol solution versus gel (21). A cohort study showed that timolol 0.5% gel forming solution was more effective than timolol 0.1% gel forming solution, suggesting that drug concentration is an important predictor for clinical responses (22). Drug frequencies also vary from twice daily to five times daily mostly based on the clinical experience of physicians (23). In our opinion, higher frequency is often associated with better efficacy but higher risk of side effects and inconvenience of administration, and three times daily is preferable for the application of timolol maleate 0.5% gel or solution. Satisfactory efficacy of this new topical timolol maleate 0.5% hydrogel is achieved, with an average VAS improvement of 76.4. Clinical responses were observed in 318 patients, and only three patients responded poorly but without further development. Currently, several clinical studies based on large sample size have been implemented. One retrospective, cohort study containing 73 patients reported that a mean improvement of 45% was observed in their study (22). The largest retrospective study to date reported over 20% with poor response demonstrated by VAS-SEV after 6–9 months of timolol therapy (13). Compared with these results, it seems that the response rate in our study is higher than other reported studies of topical timolol treatment based on large sample size, suggesting that this new formulation of timolol maleate 0.5% hydrogel might favor in improving the clinical effects.

Infantile hemangiomas have a typical growth pattern with the rapid proliferation during 5–9 postnatal months and slow regression after the first year (2). Recent studies indicate that age at treatment initiation is an important predictor of clinical responses. A retrospective study showed that better therapeutic responses were observed in patients who initiated treatments in the first 3 months (24). A prospective study by Yu et al. suggested that a higher regression rate was achieved in patients younger than 6 months old who received 4 months of topical timolol treatment (25). Our results demonstrated that higher VAS improvement was observed in groups of age ≤6 months at the start of treatment, which is consistent with previous studies. This is probably because of better inhibitory effect of beta adrenergic blockers in proliferating hemangioma cells than involuting hemangioma cells. It has been demonstrated that beta adrenergic blockers could induce vasoconstriction, endothelial cell apoptosis, and inhibition of angiogenesis through beta adrenergic signaling pathway and VEGF signaling pathway (26). Moreover, positive expression of beta adrenergic receptor-2 and VEGFR-2 has been detected in proliferating hemangioma while weakly positive expression in involuting hemangioma (26). In our opinion, the therapeutic effects of timolol at different age initiations is closely associated with the expression of beta adrenergic receptor and VEGFR at different stages, which might explain for better clinical responses during the first 1–6 months. Lesion thickness is also closely associated with clinical responses. In the study by Puttgen et al., better clinical responses were obtained in IHs < 1mm thickness compared with IHs > 1mm thickness (13). In our study, VAS score was significantly higher in IHs < 1mm thick than in IHs > 1mm thick (*P* = 0.0147). However, it should be noted that there were no significant differences in duration of treatment between ≤6 months and >6 months in the study. According to our clinical experience, it seemed that obvious clinical responses of this new topical timolol maleate 0.5% hydrogel were observed during the first 1–6 months, and subsequent improvement varied between individuals. Further well-designed prospective study should be implemented to analyze the effect of different stages of initiating topical timolol treatment on clinical responses.

Timolol is eight times as potent as propranolol by intravenous administration (27). For safety consideration, topical timolol is not suggested for treating ulcerated and mucosal IHs because of high permeability through the lesions. Compared with oral propranolol, decreased side effect is one distinct advantage of topical timolol in treating IHs. In the present study, no systemic side effects were recorded and rate of local side effect was 2% (6/321), which indicated high safety of topical timolol maleate treatment. Among six patients experiencing local side effects, four patients had pruritus after a mean duration of 1 month, manifesting as scratching the lesion after application of the hydrogel. The two patients had excoriations, but no further ulceration. All these local side effects were not significant enough to discontinue the therapy.

In conclusion, this new topical timolol maleate 0.5% hydrogel appears to be a proper candidate for treating superficial IHs with satisfactory clinical responses and mild side effects in this series. Our study provides supportive evidence and experience of topical timolol maleate in treating superficial IHs.

## Ethics Statement

The study was conducted at Department of Oral and Maxillofacial Surgery, Shanghai Ninth People’s Hospital, College of Stomatology, Shanghai Jiao Tong University School of Medicine. The study was carried out in accordance with Declaration of Helsinki. Informed consents were obtained from all parents of the patients and the study was approved by the Institute Review Board of Shanghai Ninth People’s Hospital.

## Author Contributions

HW, CL, XW, LZ, WY, JZ, LS, and XF participated in its design, searched databases, extracted and assessed studies, and helped to draft the manuscript. HW and CL wrote the manuscript. WY and JZ conceived the initial idea and the conceptualization, participated in the data extraction and analysis, and revised the manuscript. All authors read and approved the final manuscript.

## Conflict of Interest Statement

The authors declare that the research was conducted in the absence of any commercial or financial relationships that could be construed as a potential conflict of interest. The reviewers, XD and WL, and handling editor declared their shared affiliation, and the handling editor states that the process nevertheless met the standards of a fair and objective review.
